# Oxidative stress in patients with coronavirus disease and end-stage renal disease: a pilot study

**DOI:** 10.1186/s12882-024-03584-0

**Published:** 2024-05-04

**Authors:** Nam-Seon Beck, Yeonju Seo, Taesung Park, Sang‑Sin Jun, Joung-Il Im, Sae-Yong Hong

**Affiliations:** 1 Department of Pediatrics, Chung-Ang Jeil Hospital, Chungbuk, South Korea; 2 Interdisciplinary Program in Bioinformatics, Seoul National University, Seoul, South Korea; 3 Department of Statistics, Seoul National University, Seoul, South Korea; 4 Department of Neurology, Chung-Ang Jeil Hospital, Chungbuk, South Korea; 5 Department of Orthopedic Surgery, Chung-Ang Jeil Hospital, Chungbuk, South Korea; 6 Department of Nephrology, Chung-Ang Jeil Hospital, Chungbuk, South Korea

**Keywords:** COVID-19, Oxidative stress, Reactive oxygen species, Hemodialysis, End-stage renal disease

## Abstract

**Background:**

Oxidative stress, an imbalance between reactive oxygen species production and antioxidant capacity, increases in patients with coronavirus disease (COVID-19) or renal impairment. We investigated whether combined COVID-19 and end-stage renal disease (ESRD) would increase oxidative stress levels compared to each disease alone.

**Methods:**

Oxidative stress was compared among three groups. Two groups comprised patients with COVID-19 referred to the hospital with or without renal impairment (COVID-ESRD group [*n* = 18]; COVID group [*n* = 17]). The third group (ESRD group [*n* = 18]) comprised patients without COVID-19 on maintenance hemodialysis at a hospital.

**Results:**

The total oxidative stress in the COVID-ESRD group was lower than in the COVID group (*p* = 0.047). The total antioxidant status was higher in the COVID-ESRD group than in the ESRD (*p* < 0.001) and COVID (*p* < 0.001) groups after controlling for covariates. The oxidative stress index was lower in the COVID-ESRD group than in the ESRD (*p* = 0.001) and COVID (*p* < 0.001) groups. However, the three oxidative parameters did not differ significantly between the COVID and COVID-ESRD groups.

**Conclusions:**

The role of reactive oxygen species in the pathophysiology of COVID-19 among patients withESRD appears to be non-critical. Therefore, the provision of supplemental antioxidants may not confer a therapeutic advantage, particularly in cases of mild COVID-19 in ESRD patients receiving hemodialysis. Nonetheless, this area merits further research.

## Background

Diatomic oxygen can form highly reactive chemicals called reactive oxygen species (ROS), which include peroxide, superoxide, hydroxyl radical, and singlet oxygen [1]. ROS have a pathophysiologic role in regulating various cellular responses through signal transduction [2]. Generally, low levels of ROS activate pathways that promote cell survival, while high levels of ROS activate pathways that induce cell death, such as apoptosis and necrosis [3]. Mammalian cells have antioxidant enzymes to maintain the cellular redox balance,including superoxide dismutase, catalase, glutathione reductase, and peroxidase [4]. They also have non-enzyme antioxidants, such as glutathione, thioredoxin, sulfa-containing amino acids, uric acid, ascorbic acid, and vitamin E in vivo [4].

Oxidative stress (OS), the imbalance between ROS production and antioxidant capacity, is increased in patients with renal impairment due to increased oxidant activity and reduced antioxidant capacity, which worsens with renal dysfunction [5]. A source of oxidative stress in patients with end-stage renal disease (ESRD) is the uremic toxins that trigger inflammation by activating polymorph nuclear cells, IL-1β, and IL-8 [6]. Moreover, hemodialysis (HD) increases OS due to the loss of antioxidants during dialysis and the activation of white blood cells, which generate ROS [7]. OS is higher in patients with coronavirus disease (COVID-19) than in healthy individuals [8] and higher in patients with more severe forms of COVID-19 than those with mild ones or healthy controls [9].

Patients with COVID-19 and ESRD undergoing HD have a markedly increased risk of mortality and morbidity from all causes [10]. Based on this evidence, we hypothesized that combining COVID-19 and ESRD may enhance OS more than COVID-19 with normal renal function or ESRD without COVID-19.A study might be structured to validate this hypothesis by comparing the OS levels among patients with ESRD with or without COVID-19 and patients with COVID-19 without ESRD, with statistical control of potential internal confounding factors. However, few studies are available to measure oxidative stress among COVID-19 patients with ESRD, which is more valid for those employing Total oxidative stress (TOS) and total antioxidant status (TAS) assays for analysis. Thus, an independent pilot study with a small sample size was designed for preliminary data before a potential main study to validate the hypothesis.

## Methods

### Study design and participants

The study was conducted at Chung-Ang Jeil Hospital (CAJH), a secondary hospital in Jincheon County, Chungbuk Province, South Korea, and the surrounding areas, with a population of approximately 200,000.Since the World Health Organization declared COVID-19 a pandemic on January 30, 2020, South Korea has implemented an active surveillance system that works closely with private healthcare facilities. The system required all individuals with COVID-19 symptoms or epidemiological links to be tested for COVID-19. All confirmed patients with COVID-19 with ESRD on HD were sent to designated health facilities for proper medical care and isolated maintenance of HD.

Between December 21, 2021, and June 30, 2023, 1058 patients with COVID-19 (aged ≥ 18 years, males: 509, females: 549) were referred to CAJH by the Provincial Ministry of Public Health. The diagnosis of COVID-19 was based on nasopharyngeal swab positivity for severe acute respiratory syndrome coronavirus 2 (SARS-CoV-2) by polymerase chain reaction or rapid antigen testing for suspected cases that met the World Health Organization clinical criteria or had epidemiological links. Of the 1058 patients with COVID-19, 120 underwent ESRD-demanding HD.

Three groups were identified for the study. The COVID group (*n* = 17) comprised patients with confirmed COVID-19 but with normal renal function (estimated glomerular filtration rate > 60 mL/min/1.73m^2^ by the Chronic Kidney Disease Epidemiology Collaboration equation, 2021). The ESRD group (*n* = 18) comprised patients who regularly underwent HD at the CAJH for > sixmonths without evidence of SARS-CoV-2 infection. Patients with a previous SARS-CoV-2 infection within six months were excluded from this study.

In the context of a pilot study with sample sizes approximating 20 [11, 12], the recruitment strategy entailed disseminating study information to every 15th COVID-19 patient exhibiting normal renal function and every 7th patient with ESRD. Of these, 17 individuals in the COVID group and 18 in the COVID-ESRD group consented to partake in the research, providing written informed consent. Additionally, 18 ESRD patients undergoing hemodialysis (HD) at CAJH with no prior COVID-19 infection were randomly selected to participate.

### Ethical considerations

This study was approved by the Public Institutional Review Board of the Ministry of Health and Welfare of South Korea (http://irb.or.kr/menu02/summary.aspx, approval no: P01-202308–01-030). It was conducted using the principles of the Declaration of Helsinki. Written informed consent was obtained from all the participants.

### Assay

Total oxidative stress (TOS) and total antioxidant status (TAS) assays were outsourced to EONE Laboratories (Incheon, South Korea: https://www.eonelab.co.kr/institution/certi_list.asp). The commercial assay kits of TOS and TAS were from Rel Assay Diagnostics, Dusseldorf, Germany (https://www.relassay.com/products). The principles of the assay for TOS and TAS are as follows:

#### Measurement of TOS

Oxidants in the sample oxidize the ferrous ion-chelator complex to ferric ion, which forms a colored complex with chromogen in an acidic medium. The color intensity, which can be measured spectrophotometrically, is related to the sample's total amount of oxidant molecules. The assay is calibrated with hydrogen peroxide, and the results are expressed in terms of micromolar hydrogen peroxide equivalent per liter (μmolH_2_O_2_ Eq./L).

#### Measurement of TAS

The Fenton reaction generates the hydroxyl radical that reacts with the colorless substrate O-dianisidine to produce the dianisyl radical, which has a bright yellowish-brown color. Upon adding a plasma sample, the oxidative reactions triggered by the hydroxyl radicals present in the reaction mix are suppressed by the antioxidant components of the plasma. This suppression prevents color change, thereby providing an adequate measure of the total antioxidant capacity of the plasma. The assay results are expressed as mmol Trolox Eq./L.

The oxidativestress index (OSI) was calculated from the following equation:

$$\mathrm{OSI}=TOS\left(\mu mol {\mathrm H}_2{\mathrm O}_2 Equiv./L\right)/\left(TAS\times10\right)\left(mmol Trolox Equiv./L\right).$$

### Data collection

After reviewing the electronic medical records, data for the following variables were collected: age, sex, body mass index (BMI), and comorbidities of diabetes mellitus.

### Statistical analyses

Categorical variables were expressed using frequencies and percentages, while continuous variables were described using means and standard deviations. The chi-squared test was used for categorical variables to investigate the significant difference among the three groups.

Analysis of covariance (ANCOVA) was conducted to examine the group mean differences while controlling for covariates that affect oxidative stress, including age, gender, obesity, and diabetes [13–16]. Transformations were applied to TOS, TAS, and OSI due to their non-normal distribution and lack of homoscedasticity,precisely, logarithmic transformations for TAS and OSI and a square root transformation for TOS. The Shapiro–Wilk and Levene's tests confirmed the normal distribution and homogeneity of variances of the residuals, respectively [17, 18]. Following these transformations, ANCOVA was reapplied to assess the mean differences between the COVID-ESRD, COVID, and ESRD groups, adjusted for the covariates. A post-hoc Tukey's Honestly Significant Difference (HSD) test was then performed for in-depth pairwise group comparisons [19].

Statistical significance was defined as a two-sided *p*-value of < 0.05. All statistical analyses were conducted using R Statistical Software (version 4.1.2).

## Results

Demographic and clinical profiles of the patients are presented in Table 1. The prevalence of diabetes was higher among those with ESRD than those without, and the COVID group (47.7 ± 17.2 years) had a lower mean age than the ESRD (64.2 ± 16.1 years) and COVID-ESRD (62.7 ± 17.5 years) groups. However, the percentage of males and the mean BMI were not significantly different among the three groups (Table 1).

**Table 1:** Demographic and clinical profiles of the study participants

		*COVID-ESRD (n* = *18)*	*COVID (n* = *17)*	*ESRD (n* = *18)*	*p-value*
*Age* ± *SD, (years)*		62.7 ± 17.5	47.7 ± 17.2	64.2 ± 16.1	0.010
*Male, N (%)*		8 (44.4%)	11 (61.1%)	10 (58.8%)	0.555
*Diabetes, N (%)*		11 (61.1%)	4 (22.2%)	13 (76.5%)	0.004
*BMI* ± *SD (kg/m*^*2*^*)*		24.75 ± 7.0	25.84 ± 4.0	23.24 ± 4.3	0.042
*Ethnicity; N(% Korean)*		18 (100%)	18 (100%)	17 (100%)	
*COVID-19 severity, N (%)*	Mild to moderate	11 (61.1%)	16 (88.9%)	-	< 0.001
	Severe	5 (27.8%)	1 (5.6%)	-	0.038
	Mortality	2 (11.1%)	1 (5.6%)	-	0.765

*Abbreviations*: *COVID-ESRD* patients with COVID-19 with end-stage renal disease, *COVID* patients with COVID-19 with normal renal function, *ESRD* patients with renal impairment without evidence of COVID-19, *N* number, *BMI* body mass index, *SD* standard deviation, *COVID-19* coronavirus disease

**Fig. 1:**
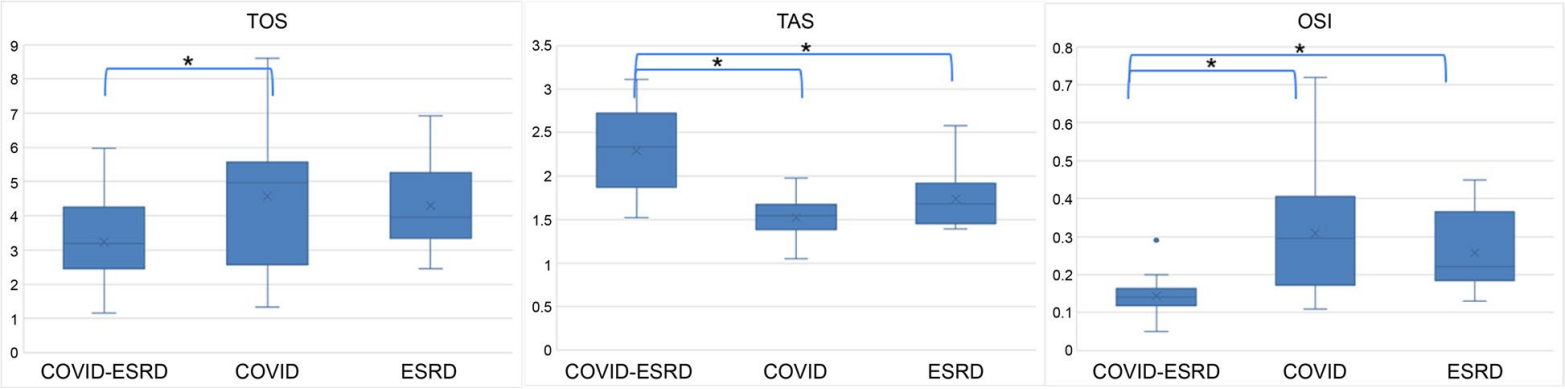
Oxidative stress parameters of the study groups. Oxidative stress index $\mathrm{OSI}=TOS\left(\mu mol{\mathrm H}_2{\mathrm O}_2\mathrm{Equiv}./\mathrm L\right)/\left(TASx\;10\right)\left(mmolTroloxEquiv./L\right)$. Abbreviations: COVID-ESRD, patients with coronavirus disease (COVID-19) with end-stage renal disease; COVID, patients with COVID-19 with normal renal function; ESRD, patients with renal impairment without evidence of COVID-19; TOS, total oxidative stress; TAS, total antioxidant status; OSI, oxidative stress index. The asterisk (*) represents those with statistical significance according to the analysis of covariance

Contrary to the hypothesis, the TOS level (μmolH_2_O_2_ Eq./L) in the COVID-ESRD group (3.24 ± 1.19) was lower than that in the COVID group (4.57 ± 2.09, *p* = 0.047;ANCOVA), while TAS (mmol Trolox Eq./L) showed significantly higher levels in the COVID-ESRD group (2.29 ± 0.49) than inthe ESRD (1.74 ± 0.33, *p* < 0.001;ANCOVA) and COVID (1.52 ± 0.23, *p* < 0.001;ANCOVA) groups after controlling the covariates including age, gender, comorbidities (diabetes), and BMI. Meanwhile, OSI (TOS/TAS × 10) was significantly lower in the COVID-ESRD group (0.14 ± 0.05) than in the ESRD (0.26 ± 0.10, *p* = 0.001;ANCOVA) and COVID (0.31 ± 0.16, *p* < 0.001;ANCOVA) groups. However, the three oxidative parameters (TOS, TAS, and OSI) did not differ statistically between the COVID and the ESRD groups (Table 2, Fig. 1).

**Table 2:** Oxidative values in the three study groups

	*COVID-ESRD (n* = *18)*	*COVID (n* = *17)*	*ESRD (n* = *18)*	*COVID-ESRD vs. ESRD (p-value)*	*COVID-ESRD vs. COVID (p-value)*	*ESRD vs. COVID (p-value)*
*TOS* ± *SD (μmol H*_*2*_*O*_*2*_*Eq./L)*	3.24 ± 1.19	4.57 ± 2.09	4.31 ± 1.31	0.786	0.047	0.979
*TAS* ± *SD (mmol Trolox Eq./L)*	2.29 ± 0.49	1.52 ± 0.23	1.74 ± 0.33	< 0.001	< 0.001	0.156
*OSI* ± *SD (TOS/10* × *TAS)*	0.14 ± 0.05	0.31 ± 0.16	0.26 ± 0.10	0.001	< 0.001	0.281

*Abbreviations*: *COVID-ESRD* patients with COVID-19 with end-stage renal disease, *COVID* patients with COVID-19 with normal renal function, *ESRD* patients with renal impairment without evidence of COVID-19, *TOS* total oxidative stress, *TAS* total antioxidant status, *SD* standard deviation, *OSI* oxidative stress index, *COVID-19* coronavirus disease

*p-value* is estimated by Tukey’s Honestly Significant Difference test for posthoc analysis following the ANCOVA, allowing for comprehensive pairwise comparisons among these groups

## Discussion

Oxidative stress markers, including TOS, are higher in COVID-19 cases than healthy individuals [8]. Patients with ESRD are also characterized by enhanced oxidative stress [20, 21]. Moreover, renal replacement therapy often accelerates prooxidant activity [7]. However, contrary to expectations, the present study showed that TOS was lower in the COVID-ESRD group than in the COVID group (Table 2, Fig. 1).

Uremia-induced immunosuppression, characterized by reduced synthesis of inflammatory cytokines following antigen stimulation [22] and B-cell lymphopenia in ESRD patients [23], may collectively serve as a suppressive mechanism against the generation of ROS. Given these, we cautiously hypothesize that within the uremic environment, the impaired immune response to COVID-19 could diminish stimulatory factors involved in ROS generation [24].

In general, for patients with ESRD, antioxidant status often deteriorates [25]. Altered dietary restrictions and preferences may exaggerate the depletion of antioxidant defense mechanisms, such as low levels of vitamins C and E, reduced selenium levels, decreased function of the glutathione scavenging system, and loss of vitamins during HD [26, 27]. However,the present study revealed a prominent increase in TAS in the COVID-ESRD group (Table 2, Fig. 1). Typically, exposure to pro-oxidants triggers the activation of antioxidant defense mechanisms. The activation of antioxidative enzymes may be attributed to decreased tACE2 levels upon viral entry into cells, alongside increased biologically active sACE2 levels due to heightened ADAM-17 activity [28, 29]. Augmented sACE2 may stimulate nuclear factor erythroid 2–related factor (Nrf2) [30], which in turn triggers the transcription of antioxidant genes such as superoxide dismutase, catalase, and glutathione peroxidase [31, 32].

Multiple factors may augment the antioxidative response in the COVID-ESRD group. Initially, the clinical severity observed in this study was predominantly mild, which contrasts with the cases reported in other researches. Evidence indicates a correlation between reduced antioxidant enzyme expression in patients with severe COVID-19 [33].

Furthermore, the pro-oxidant stress levels in this patient group might not have reached the threshold necessary to chelate antioxidants produced by the viral antigen. Additionally, it is noteworthy that, based on anecdotal evidence, a minority of the participants within the COVID-ESRD group might have commenced antioxidant therapy prior to their inclusion in the study, a detail that was not documented within the research parameters.Interestingly, a cross-sectional study also showed that serum total antioxidant capacity increased gradually along with the deterioration of renal function in patients with the stages of renal failure [34].

Oxidative stress is both a cause and a consequence of the pathophysiology of the infectious process [35]. In the early stages of viral infections, administering proinflammatory cytokines, which precede reactive ROS, can assist in managing the virus before adverse immune consequences manifest [36]. Considering that most COVID-19 cases within the COVID-ESRD group present with early-stage infection (mild to moderate), the attenuated oxidative stress observed may not contribute favorably to the prognosis of COVID-19 in individuals with renal dysfunction.

In a comprehensive, multinational, randomized clinical trial [37], the administration of vitamin C to hospitalized COVID-19 patients did not improve organ support-free days or hospital survival. Moreover, vitamin C was associated with a worsening of both outcomes in critically ill and non-critically ill patients. Considering that the sequence of physiological responses induced by oxidative stress could be a significant factor in the worsening of the host's condition [38], additional investigationis warranted for this unexpected findingin COVID-19 patients with ESRD.

This study has a few limitations. The primary challenge lies in quantifying oxidative stress within clinical medicine, given the intricate nature of the procedure and the several elements involved, encompassing both oxidants and antioxidants [39]. Although the statistical analysis was designed to control some internal factors affecting oxidative stress (age, sex, obesity, and comorbidities), the analysis has its inborn limitations caused by its small sample size. Furthermore, the study did not control for most external factors (physical activity, diet, medications, climate, etc.) affecting OS. Another limitation is the unavailability of control data in the general population, i.e., participants with normal renal function and without COVID-19 infection. Thus, a larger sample size requires further investigations with controlling internal and external confounding factors. Despite the limitations, this is the first study on oxidative stress parameters in patients with COVID-19 and ESRD on HD.

## Conclusions

The role of reactive oxygen species in the pathophysiology of COVID-19 among patients withESRD appears to be non-critical. Therefore, the provision of supplemental antioxidants may not confer a therapeutic advantage, particularly in cases of mild COVID-19 in ESRD patients receiving hemodialysis. Nonetheless, this area merits further research.

## Data Availability

The data presented in this study are available on request from the corresponding author (syhong0526@gmail.com).

## Abbreviations

ANCOVA: Analysis of covariance
BMI: Body mass index
CAJH: Chung-Ang Jeil Hospital
COVID-19: Coronavirus disease
ESRD: End-stage renal disease
HD: Hemodialysis
Nrf2: Nuclear factor erythroid 2–related factor
OS: Oxidative stress
OSI: Oxidative stress index
ROS: Reactive oxygen species
SARS-CoV-2: Severe acute respiratory syndrome coronavirus 2
TAS: Total antioxidant status
TOS: Total oxidative stress
